# In Vitro Cone Beam CT Analysis of the Efficacy of Three Supplementary Steps In the Removal of Obturation Material in Endodontic Retreatment

**DOI:** 10.7759/cureus.62285

**Published:** 2024-06-13

**Authors:** Krishna Priya N, Priya R, Sonu Ravindran, Jis John, Suraj U, Fazalu Rahman

**Affiliations:** 1 Conservative Dentistry and Endodontics, Malabar Dental College and Research Center, Malappuram, IND

**Keywords:** passive ultrasonic irrigation, cbct, eddy, xp-endo finisher r, d-race retreatment file, root canal retreatment

## Abstract

Objectives: To evaluate and compare the efficacy of XP-endo Finisher R (FKG Dentaire, Le Locle, Neuchatel, Switzerland), EDDY (VDW Dental, Munich, Germany), and passive ultrasonic irrigation (PUI) as supplementary steps following the D-RaCe retreatment file system (FKG Dentaire) in the removal of root canal obturation material using cone beam CT.

Materials and methods: A total of 45 two-rooted permanent maxillary first premolars were selected. Following access preparation, cleaning, and shaping with Hero Shaper (Micro Mega, Besançon, BFC, France) rotary file up to 25/04%, thermoplasticized obturation was performed with TotalFill BC sealer (FKG Dentaire) and gutta-percha. The specimens were subjected to routine retreatment using the D-RaCe retreatment file system. Cone beam computed tomography (CBCT) and volumetric analysis were performed before and after this procedure. The samples were divided into group A (XP-endo Finisher R: n=15), group B (EDDY: n=15), and group C (PUI: n=15). Finally, a third CBCT was taken and a volumetric analysis was done. Statistical analysis was done using SPSS Statistics version 26.0 (IBM Corp., Armonk, NY, USA).

Results: The lowest mean residual volume of obturation material was seen with XP-endo Finisher R (1.6 mm^3^), followed by PUI (1.7 mm^3^). The EDDY showed the least efficiency in complete debridement of the root canals (3.6 mm^3^). This difference in values was statistically significant.

Conclusion: The XP-endo Finisher R and PUI showed superior performance than EDDY in the removal of remaining obturation material from the root canal system after retreatment with the D-RaCe retreatment file system. However, none of the systems were able to completely remove the root canal obturation materials.

## Introduction

Endodontic therapy is riddled with challenges in combating infection in the root canal system and has been reported to have a success rate between 85% and 95% [1]. Despite the high success rates and predictability of endodontic therapy, failures can nevertheless happen due to persistent infections or recontamination of the root canal system after endodontic intervention [2].

Non-surgical retreatment is considered the primary treatment option in teeth with post-treatment disease and, when performed successfully, helps in regaining the health of periapical and periradicular tissues [3]. To achieve a successful outcome for endodontic retreatment, coronal disassembly of prostheses such as crowns and bridges and retrieval of cemented, bonded, or mechanically locked posts followed by total removal of root canal obturation materials like gutta-percha and sealers is necessary [4].

The different approaches for removing obturation materials include heated instruments, manual instruments with or without chemical solvents, Gates-Glidden drills, and rotary instruments or reciprocation systems [5]. The D-RaCe (FKG Dentaire, Le Locle, Neuchatel, Switzerland) is a rotary retreatment file system consisting of two files, D1 and D2, of varying tip size and taper, which has been reported as most effective among popular retreatment systems [6,7]. Unfortunately, gutta-percha and sealer residue persist on canal walls with most techniques [8,9]. Therefore, supplementary methods and techniques specifically designed to improve the removal of residual obturation materials have been proposed.

Passive ultrasonic irrigation (PUI) using various irrigants has been recommended to facilitate thorough cleaning and removal of root canal contents in cases of retreatment [10]. The XP-endo Finisher R (FKG Dentaire) is a specifically designed file system to effectively clean root canals during retreatment procedures. Another system that has been recommended to supplement root canal debridement in cases of retreatment is EDDY (VDW Dental, Munich, Germany), consisting of a sonically activated polyamide tip. The present study was conceived as a comparative evaluation of the efficacy of these three supplementary systems in the total removal of obturation material in retreatment cases, as evaluated by cone beam computed tomography (CBCT). The null hypothesis tested was that there is no significant difference between the efficacy of XP-endo Finisher R, EDDY, and PUI in total removal of obturation material from root canals in retreatment cases.

## Materials and methods

A total of 45 sound caries-free double-rooted maxillary premolar teeth with a closed apex were selected. The study was conducted with the approval of the Institutional Ethics Committee of Malabar Dental College and Research Center, Malappuram, KL, IND (approval no. IEC/04/CONS-B/MDC/2020) for the collection and use of maxillary premolars extracted for orthodontic purposes. Teeth with root caries, complex internal anatomy, calcified canals, or thin curved roots were excluded. All the collected teeth were stored according to Occupational Safety and Health Administration (OSHA) regulations.

Specimens were decoronated with a diamond disc at the cementoenamel junction under copious water cooling, retaining a root length of 14 ± 2 mm from the apex. The access cavity was prepared, and the working length was determined by inserting an International Organization Standard (ISO) #10K file (Mani Inc., Takanezawa, Tochigi, JPN) until it was just visible at the apical foramen and then reducing 1mm. Cleaning and shaping were performed with the Hero Shaper rotary file (Micro Mega, Besançon, BFC, France) up to size ISO #25 as per standard protocol. Irrigation was done with 1 ml of 5.25% sodium hypochlorite (Asian Acrylates, Mumbai, MH, IND) between each instrument. The removal of debris and smear layer was performed with 5 ml 7% maleic acid (Nice Chemicals, Cochin, KL, IND) followed by 1 ml 5.25% sodium hypochlorite; 2 ml distilled water was used as the final flush, and in between the above irrigants. After drying with paper points, the root canals were coated with a calcium silicate-based bioceramic sealer TotalFill BC sealer (FKG Dentaire) using Lentulospiral (Mani Inc.). The master cone was inserted 1 mm short of the working length. The coronal part of the gutta-percha was cut with an obturation pen (Denjoy iFill GP Obturation System, Changsha City, HN, CHN) until 3 mm to 4 mm gutta-percha remained at the apical one-third, and then vertical compaction was done with a hand plugger. After this, the coronal two-thirds were obturated with warm gutta-percha using an obturation gun (Denjoy) [11]. The CBCT images of the obturated teeth were taken with ProMax 3D ProFace CBCT machine (Planmeca, Helsinki, Finland) with a high artefact removal algorithm, and the volumetric evaluation of the amount of obturation material in each canal was estimated using Romexis software (Planmeca). All specimens were then kept at 37ºC and 100% humidity for two weeks in an incubator to simulate in vivo conditions. The materials used in this study are listed in Table 1.

**Table 1:** List of materials GP: Gutta-percha

Material	Product	Specification	Company
Rotary files	Hero Shaper	4%, #25	MicroMega, France
Retreatment files	D-RaCe	DR1, DR2	FKG Dentaire, Switzerland
Supplementary systems	XP-endo Finisher R		FKG Dentaire, Switzerland
	EDDY system		VDW Dental, Germany
	U-file	#25	Mani Inc., Japan
Gutta-percha	Denjoy iFill GP Obturation system		Denjoy, China
Bioceramic sealer	TotalFill BC		FKG Dentaire, Switzerland
Irrigants	Sodium hypochlorite	5.25%	Asian Acrylates, India
	Maleic acid	7%	Nice Chemicals, India
GP solvent	Carvene		Prevest DentPro, India

Retreatment was initiated in the specimens using gutta-percha solvent (Carvene; Prevest DenPro, Jammu, India) and the D-RaCe (DR) rotary retreatment file system. The coronal third of the root filling was removed using the DR1 instrument (#30/10 taper) operated at 1000 rpm. The DR2 instrument (#25/04 taper) was used with light apical pressure at 800 rpm until the working length was reached [12]. A second CBCT was taken to determine the residual amount of obturation material at this stage. The specimens were then randomly assigned to one of three groups, i.e., group A treated with XP-endo Finisher R (n=15); group B treated with EDDY (n=15); and group C treated with PUI (n=15).

**Figure 1:**
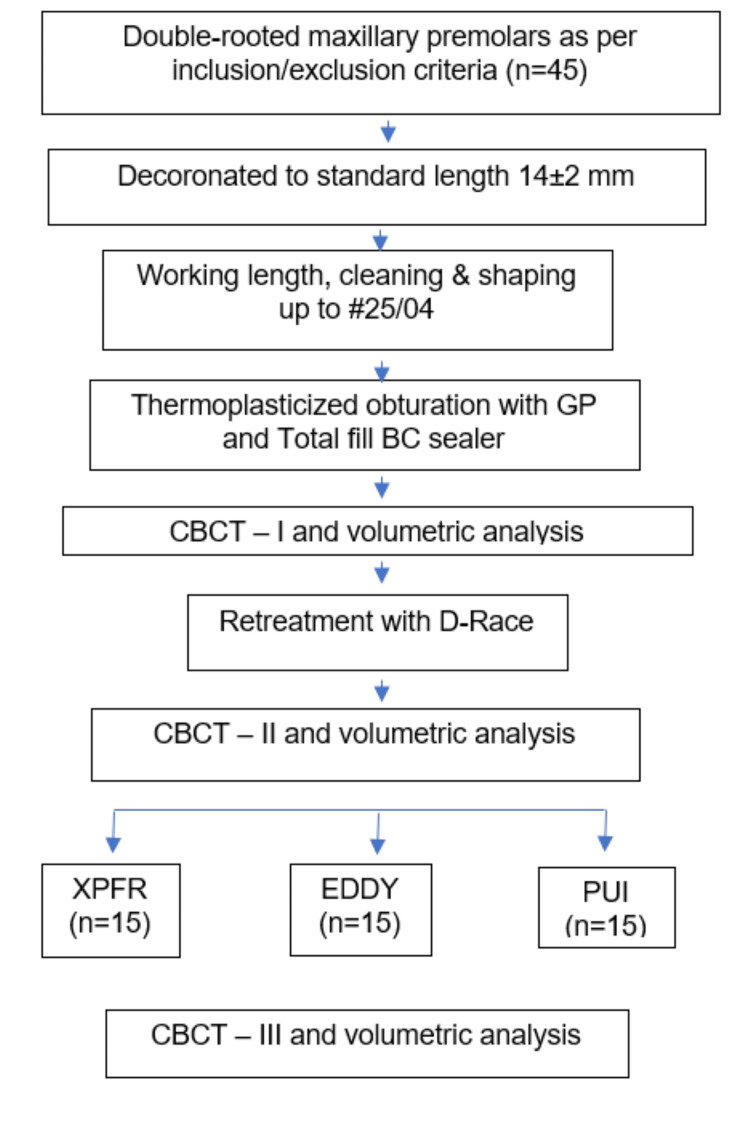
Methodology flowchart GP: Gutta-percha, CBCT: Cone beam computed tomography, XPFR: XP-endo Finisher R, PUI: Passive ultrasonic irrigation

In group A, the XP-Endo Finisher R file was inserted in the root canals and then activated (1000 rpm and 1 Ncm) 1mm short of the apex using slow and gentle 7 mm to 8 mm lengthwise movements for 30 seconds. The specimens in group B were treated with the EDDY sonic tip inserted up to 1 mm short of the working length and activated at 6000 Hz/160 µm with in-and-out movements of 5 mm amplitude. In group C, the E1 ultrasonic tip with U-file size 25 (Mani Inc.) was mounted on an ultrasonic unit (Woodpecker, Guilin, GX, CHN) set at a frequency of 30 kHz, inserted in the root canal up to 2 mm short of the working length, and activated for 30 seconds [13]. This procedure was repeated two times [14]. These supplementary steps were done using 5 ml of 7% maleic acid as the irrigant. A final CBCT scan was taken to assess the volume of residual root canal obturation material, if any. The methodology flow chart is shown in Figure 1.

## Results

The data were subjected to statistical analysis using SPSS Statistics version 26.0 (IBM Corp., Armonk, NY, USA). The level of significance was set at p<0.05. Descriptive statistics was performed to assess the mean and standard deviation of the respective groups. The normality of the data was assessed using the Shapiro-Wilkinson test. Since the data followed a normal distribution, parametric tests were used for the analysis. A one-way ANOVA test followed by Tukey’s honestly significant difference (HSD) test was used to check the difference between the three groups.

**Figure 2:**
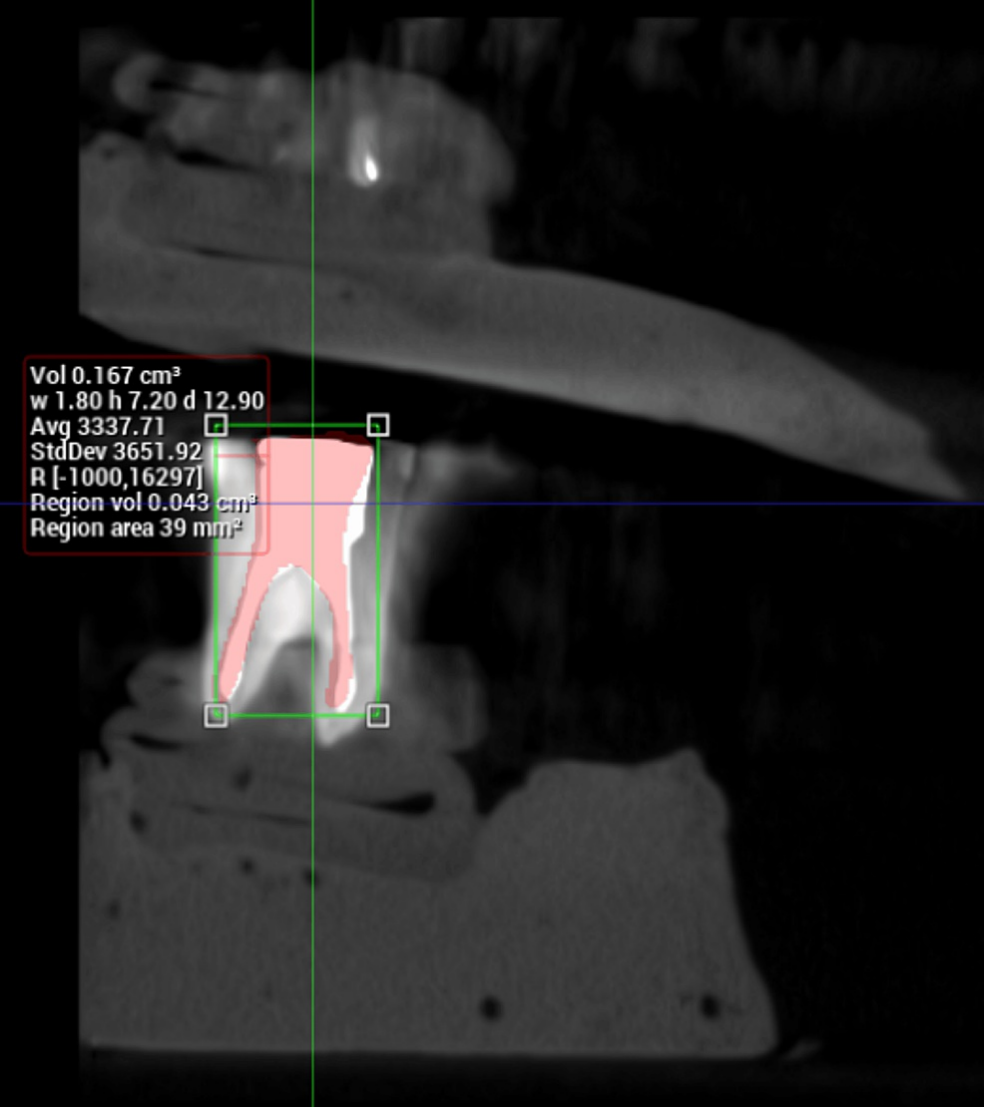
Calculation of volume from CBCT image taken after obturation CBCT: Cone beam computed tomography

The volume of obturation material in each specimen was obtained from the first CBCT image that was taken immediately after obturation (Figure 2). These values ranged from 43 mm^3^ to 119 mm^3^ and the mean volume of obturation material was 72 mm^3^.

**Figure 3:**
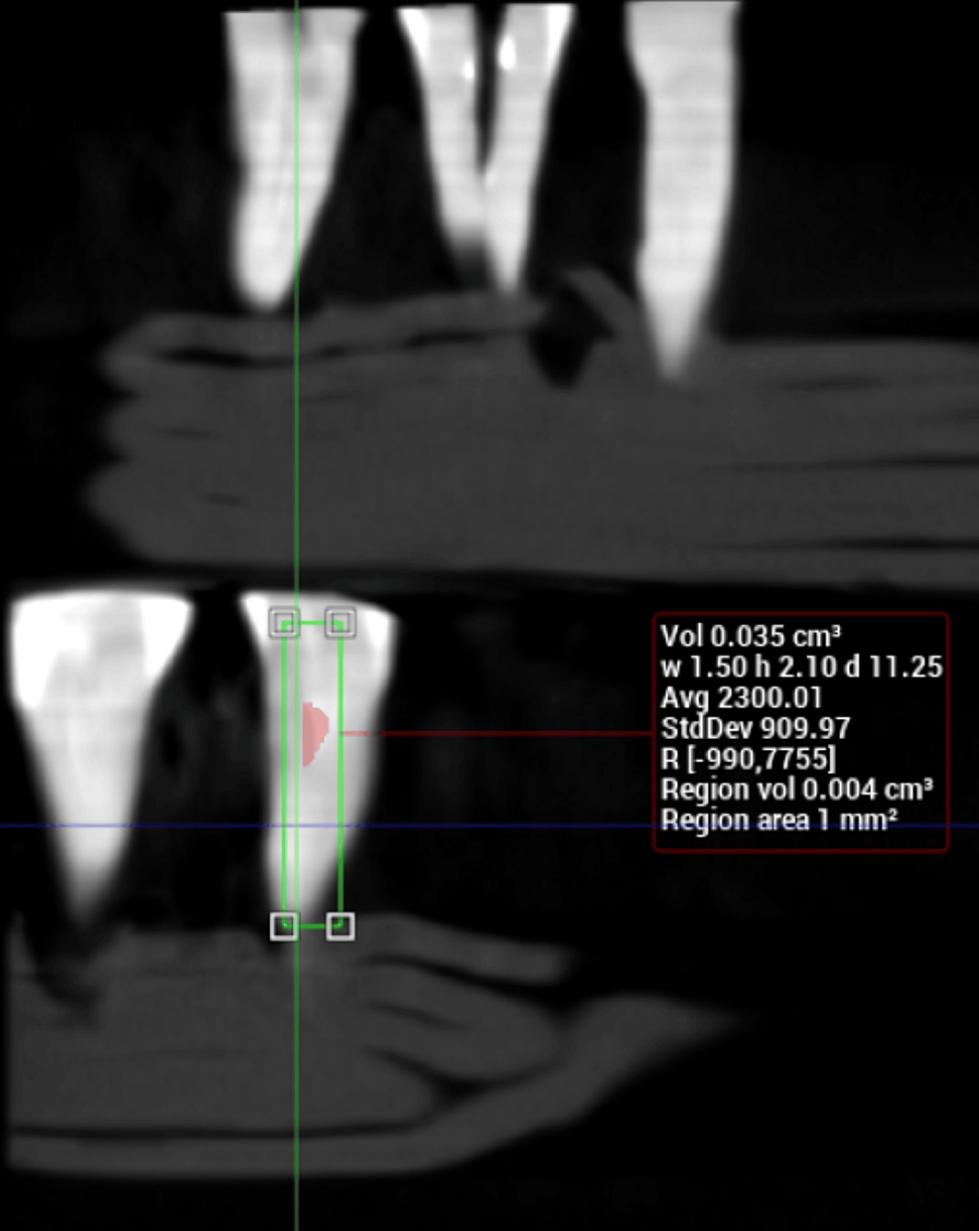
Calculation of volume from CBCT image after retreatment with D-RaCe CBCT: Cone beam computed tomography

The second CBCT image revealed the residual volume of obturation material after retreatment with the D-RaCe file system, the highest value being 17 mm^3^ (Figure 3). Only one specimen showed a near-total removal of obturation material, and the mean volume was calculated as 8.1 mm^3^. Thus, retreatment with the D-RaCe file system alone resulted in a residual obturation material volume of 11.25%.

**Figure 4:**
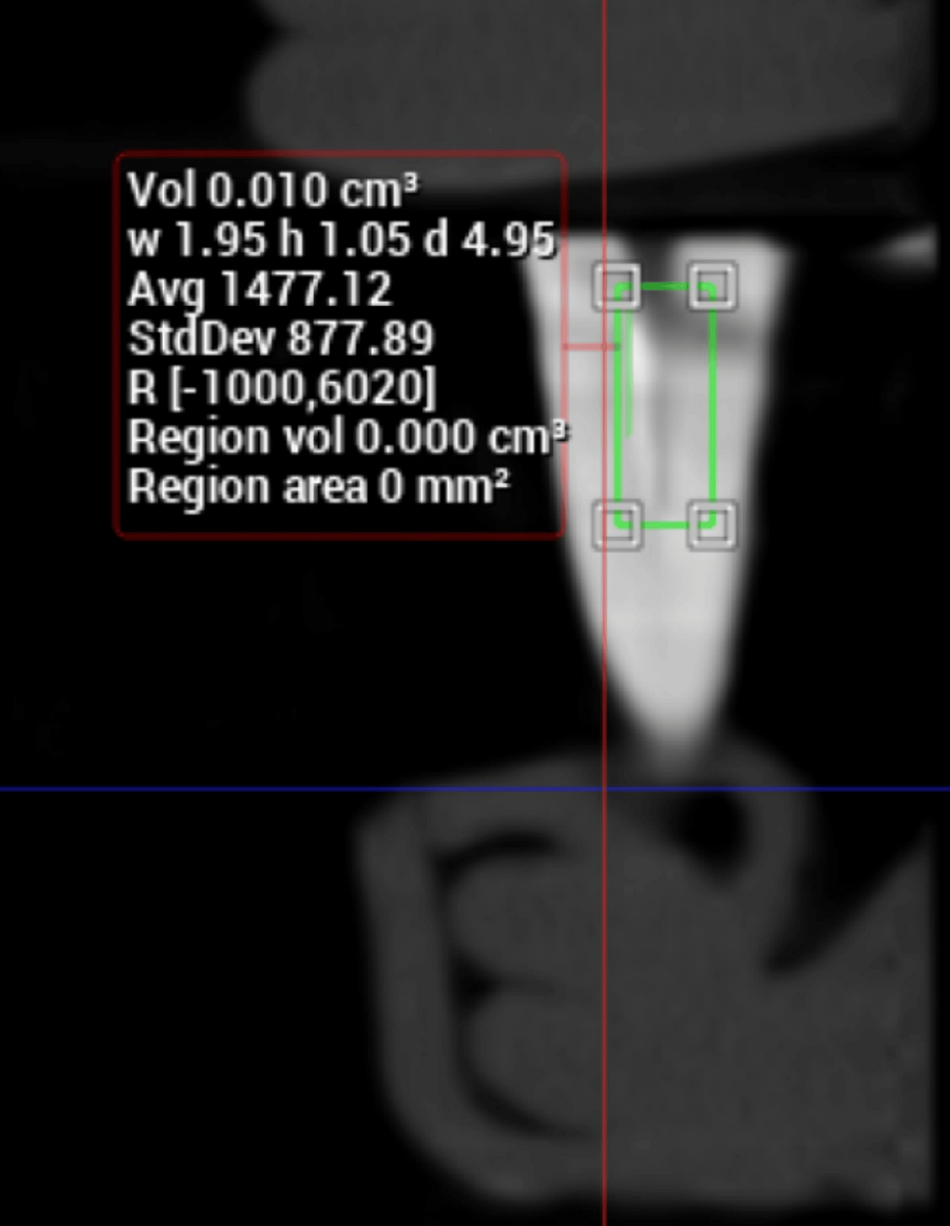
Calculation of volume from CBCT image after supplementary step CBCT: Cone beam computed tomography

The mean volume of residual obturation material after each supplementary step is given in Table 2. The values for group A (XP-endo Finisher R) ranged up to a maximum of 5 mm^3^, and this high value was seen in a single specimen. Twelve specimens showed values in the range 1 mm^3^ to 2 mm^3^ with two specimens exhibiting total removal of obturation material (Figure 4). In the specimens of group C (PUI), the values were similar to group A. The majority of the specimens showed values ranging from 1 mm^3^ to 2 mm^3^, two had a value of 4 mm^3^, and one showed total removal.

**Table 2:** Mean volume (cm3) of residual obturation material after each supplementary step XPFR: XP-endo Finisher R, PUI: Passive ultrasonic irrigation

	Group A (XPFR)	Group B (EDDY)	Group C (PUI)	p-value	Post hoc	
Mean	0.001	0.003	0.001	0.0001*	A vs. B	0.0001*
SD	0.001	0.001	0.001		A vs. C	0.99
					B vs. C	0.0001*

In comparison, larger volumes of residual obturation material were seen with group B (EDDY). Among these specimens, the highest value was 7 mm^3^, with most specimens showing values ranging from 2 mm^3^ to 4 mm^3^. Only two specimens showed a value of 1 mm^3^ and no specimen could claim a total removal of obturation material. Two-tailed ANOVA test showed that this difference between groups was statistically significant.

Post hoc analysis for pair-wise comparison between each group was performed (Table 2). The analysis showed that the difference in values between groups A and B, as well as between groups B and C was statistically significant (p=0.001). However, the difference between groups A and C was not significant (p=0.99).

## Discussion

A successful root canal retreatment requires the removal of all existing obturation material [15]. Any residual obturation material left behind can act as a source of contamination, leading to periapical inflammation and delayed healing [16]. Therefore, the amount of residual obturation material is said to have a direct impact on the long-term prognosis of a tooth that is undergoing root canal retreatment [17].

Two-rooted maxillary premolars having oval canals are difficult to clean and shape and often pose a challenge for the clinician to completely remove obturation materials during retreatment [18]. For this reason, the present study was conducted in two-rooted maxillary premolars. The thermoplasticized obturation technique has been reported to create a better adaptation of gutta-percha to root canal walls, improve the degree of homogeneity, and provide optimum coronal and apical seals as compared to cold lateral condensation [19,20]. Therefore, this technique of obturation was adopted in our study. Along with this, TotalFill BC, a tricalcium silicate-based sealer claimed to have improved sealing ability, biocompatibility, antibacterial property, and biomineralization capacity, was used.

Assessment of the quality of root canal obturation as well as the amount of obturation material removed during retreatment is inaccurate with periapical radiographs due to projection geometry issues, superimposition of adjacent structures, as well as anatomic noise from surrounding structures [21]. Cone beam CT is specifically designed to reproduce 3D images of the maxillofacial region [22]. This type of imaging provides accurate data about maxillofacial structures, especially teeth, and the associated structures, with a reduced effective dose of radiation as compared to medical CT [22,4,21].

The D-RaCe retreatment system comprises two files of varying lengths and tapers. The DR1 has a tip size of 30 and a taper of 10%, which also has a cutting tip for easy penetration into the root canal filling material. The DR2 consists of a tip size of 25 and a taper of 4%, which is used for cleaning the apical two-thirds [23]. In the present study, the application of this file system alone was seen to be ineffective in the total removal of obturation material, with a residual volume of above 10%. This is in accordance with the results reported by Madani et al., who compared the efficacy of D-RaCe, ProTaper retreatment files (Dentsply Sirona, Charlotte, NC, USA), and hand filing techniques [24].

The EDDY is a recently introduced sonic device for irrigant activation and is a polymer rod-based system. The polyamide tip receives vibration from the air scaler, and this leads to an oscillating motion of high amplitude. This high-amplitude motion is claimed to cause cavitation and acoustic streaming, thus leading to effective debridement of the root canal system during retreatment [25]. In comparative studies between EDDY and other irrigant activation systems, including Endoactivator (Dentsply Sirona), the former has been reported to exhibit superior performance in terms of debridement and smear layer removal [25,26].

In the present study, the volume of obturating material removed by D-RaCe followed by EDDY was significantly lower than XP-endo Finisher R. This result is supported by research conducted by De Deus et al., Uzunoglu-Ozyurek et al., and Hassan et al., where they established the superior efficacy of XP-endo Finisher R in removing the remaining obturation material [14,27,28]. The XP-endo Finisher R is a file system that utilizes Max-Wire technology. This treated alloy undergoes a phase transformation from martensitic to austenitic phase almost near body temperature at 35 degrees Celsius, resulting in a semicircular shape for the XP-endo file system. This leads to an eccentric rotational motion, thereby ensuring thorough debridement [27].

In our study, PUI was also seen to debride more efficiently than EDDY, the difference being statistically significant. Passive ultrasonic irrigation produces high-frequency ultrasonic waves during activation that cause cavitation as well as acoustic streaming [28]. The superior efficacy of PUI in the present study may be attributed to this resultant high energy. After a detailed analysis of the results of the present study, the null hypothesis was rejected, as the difference in efficacy between the supplementary systems was statistically significant.

Limitations and strengths

Although the study was in vitro, all measures were taken to simulate intraoral conditions, but it may not be possible to directly extrapolate the results of the present study to a clinical situation. This limitation is consistent with all in vitro studies, and the results of the present study encourage an in vivo approach for more clinically applicable outcomes. Since a thoroughly researched methodology and recommended protocol were adhered to, the study, despite its in vitro nature, provides valuable information about the efficacy of the studied supplementary systems.

## Conclusions

Neither the retreatment file system alone nor a combination with the three supplementary systems was able to completely remove the obturation material from the root canals of all except three specimens. Although the residual volume of obturation material was low (mean volume = 1.6 mm^3^), the effect of these remnants on the prognosis of retreatment cases cannot be precluded. This leaves a lacuna for further research to develop a mechanism to debride the root canal system completely of all obturation material in cases of retreatment.

However, the results of the present study clearly indicate that the use of a supplementary step such as PUI or XP-endo Finisher R can significantly reduce the volume of residual obturation material. Therefore, within the limitations of the study, both PUI using maleic acid irrigant as well as the XP-endo Finisher R system may be recommended in cases of retreatment to facilitate greater removal of obturation material and ensure a more favorable prognosis.

## References

[1] Swartz DB, Skidmore AE, Griffin JA Jr (1983). Twenty years of endodontic success and failure. J Endod.

[2] Salehrabi R, Rotstein I (2004). Endodontic treatment outcomes in a large patient population in the USA: an epidemiological study. J Endod.

[3] Crozeta BM, de Sousa-Neto MD, Leoni GB, Mazzi-Chaves JF, Silva-Sousa YTC, Baratto-Filho F (2016). A micro-computed tomography assessment of the efficacy of rotary and reciprocating techniques for filling material removal in root canal retreatment. Clin Oral Investig.

[4] Neelakantan P, Grotra D, Sharma S (2013). Retreatability of 2 mineral trioxide aggregate-based root canal sealers: a cone-beam computed tomography analysis. J Endod.

[5] Bueno CE, Delboni MG, de Araújo RA, Carrara HJ, Cunha RS (2006). Effectiveness of rotary and hand files in gutta-percha and sealer removal using chloroform or chlorhexidine gel. Braz Dent J.

[6] Kontogiannis TG, Kerezoudis NP, Kozyrakis K, Farmakis ET (2019). Removal ability of MTA-, bioceramic-, and resin-based sealers from obturated root canals, following XP-endo® Finisher R file: an ex vivo study. Saudi Endod J.

[7] Karamifar K, Mehrasa N, Pardis P, Saghiri MA (2017). Cleanliness of canal walls following gutta-percha removal with hand files, race and race plus XP-endo Finisher instruments: a photographic in vitro analysis. Iran Endod J.

[8] Crozeta BM, Silva-Sousa YT, Leoni GB, Mazzi-Chaves JF, Fantinato T, Baratto-Filho F, Sousa-Neto MD (2016). Micro-computed tomography study of filling material removal from oval-shaped canals by using rotary, reciprocating, and adaptive motion systems. J Endod.

[9] de Siqueira Zuolo A, Zuolo ML, da Silveira Bueno CE, Chu R, Cunha RS (2016). Evaluation of the efficacy of TRUShape and Reciproc file systems in the removal of root filling material: an ex vivo micro-computed tomographic study. J Endod.

[10] de Souza DS, S Silva AS, Ormiga F, Lopes RT, Gusman H (2021). The effectiveness of passive ultrasonic irrigation and the easy-clean instrument for removing remnants of filling material. J Conserv Dent.

[11] Yulianda SS, Usman M, Margono A (2017). Density comparison of root canal obturation at apical one-third between single cone and downpack-backfill techniques using polidimetylsiloxane sealer. J Phys Conf Ser.

[12] Rödig T, Hausdörfer T, Konietschke F, Dullin C, Hahn W, Hülsmann M (2012). Efficacy of D-RaCe and ProTaper universal retreatment NiTi instruments and hand files in removing gutta-percha from curved root canals — a micro-computed tomography study. Int Endod J.

[13] Agrawal S, Boruah LC, Rajkumar B, Singh G (2019). Ultrasonic tips in endodontics — a review of literature. IP Indian J Conserv Endod.

[14] De-Deus G, Belladonna FG, Zuolo AS (2019). XP-endo Finisher R instrument optimizes the removal of root filling remnants in oval-shaped canals. Int Endod J.

[15] Ng YL, Mann V, Gulabivala K (2011). A prospective study of the factors affecting outcomes of nonsurgical root canal treatment: part 1: periapical health. Int Endod J.

[16] Endo MS, Ferraz CC, Zaia AA, Almeida JF, Gomes BP (2013). Quantitative and qualitative analysis of microorganisms in root-filled teeth with persistent infection: monitoring of the endodontic retreatment. Eur J Dent.

[17] Yang X, Lan J, Ji M, Tsauo C, Gao Y, Zou L (2022). Assessment of the effectiveness of supplementary methods for residual filling material removal using micro-computed tomography: a systematic review and meta-analysis of in vitro studies. Eur Endod J.

[18] Abarca J, Zaror C, Contreras W, Jadue S, Olguin C, Steinfort K, Monardes H (2018). Morphology of the physiological apical foramen of maxillary premolars. Int J Morphol.

[19] O'Sullivan SM, Hartwell GR (2001). Obturation of a retained primary mandibular second molar using mineral trioxide aggregate: a case report. J Endod.

[20] Bapna R, Makandar SD, Dordi J, Bapna PA, Karobari MIA (2019). Evaluation of voids in thermoplastisized obturation using MTA Fill apex sealer. Int J Sci Res.

[21] Yilmaz F, Sonmez G, Kamburoglu K, Koc C, Ocak M, Celik HH (2019). Accuracy of CBCT images in the volumetric assessment of residual root canal filling material: effect of voxel size. Niger J Clin Pract.

[22] Angelopoulos C, Scarfe WC, Farman AG (2012). A comparison of maxillofacial CBCT and medical CT. Atlas Oral Maxillofac Surg Clin North Am.

[23] Garg A, Nagpal A, Shetty S, Kumar S, Singh KK, Garg A (2015). Comparison of time required by D-RaCe, R-Endo and Mtwo instruments for retreatment: an in vitro study. J Clin Diagn Res.

[24] Madani ZS, Simdar N, Moudi E, Bijani A (2014). CBCT evaluation of the root canal filling removal using D-RaCe, ProTaper retreatment kit and hand files in curved canals. Iran Endod J.

[25] Haupt F, Meinel M, Gunawardana A, Hülsmann M (2020). Effectiveness of different activated irrigation techniques on debris and smear layer removal from curved root canals: a SEM evaluation. Aust Endod J.

[26] Urban K, Donnermeyer D, Schäfer E, Bürklein S (2017). Canal cleanliness using different irrigation activation systems: a SEM evaluation. Clin Oral Investig.

[27] Uzunoglu-Özyürek E, Küçükkaya Eren S, Karahan S (2021). Contribution of XP-Endo files to the root canal filling removal: a systematic review and meta-analysis of in vitro studies. Aust Endod J.

[28] Hassan R, Elzahar S (2022). Cleaning efficiency of XP Finisher, XP Finisher R and passive ultrasonic irrigation following retreatment of teeth obturated with TotalFill HiFlow bioceramic sealer. Eur Endod J.

